# Conversations About Death and Dying, End-of-Life Care Plans and Preferences Between Aging Parents and Adult Children

**DOI:** 10.1093/geroni/igaa057.1345

**Published:** 2020-12-16

**Authors:** Hyo Jung Lee, Jacobbina Jin Wen Ng

**Affiliations:** Nanyang Technological University, Singapore, Singapore

## Abstract

This study aims to investigate whether attitude and perception on late-life death and dying, end-of-life care plans and preferences could be better understood from current values shared between aging parents and their adult children in the multi-cultural city-bound country, Singapore. We are in the process of interviewing 20 aging parent-adult child dyads. Up to date, six semi-structured interviews were completed and transcribed. We performed Content analysis to analyze the transcripts. Preliminary findings showed that both aging parents and adult children rarely discussed this issue, although parents had their own plans or preferences. The major barriers against open conversations about death and dying of aging parents include: the perception of not-yet time to talk about this issue (without knowing when the right time is) and tendency to have conversations about death in tandem with finances, but not death itself. Although specific end-of-life care plans or arrangements were not thought out thoroughly, aging parents expressed a high level of trust and reliance on close family members’ decisions regarding their end-of-life care. They tended to agree on joint decision-making process within family, even though adult children had no or unmatched ideas about their aging parents’ end-of-life wishes. This did not necessarily align with previous findings in Western countries, underscoring individuals’ control over their own death and dying process. Open conversation within family, family-involved advance care planning, or joint decision-making processes may be warranted to promote quality of life and death in older Singaporeans and well-being of their family members of all ages.

